# CCR4^+^ Skin-Tropic Phenotype as a Feature of Central Memory CD8^+^ T Cells in Healthy Subjects and Psoriasis Patients

**DOI:** 10.3389/fimmu.2020.00529

**Published:** 2020-04-03

**Authors:** Fabio Casciano, Marco Diani, Andrea Altomare, Francesca Granucci, Paola Secchiero, Giuseppe Banfi, Eva Reali

**Affiliations:** ^1^Department of Morphology, Surgery and Experimental Medicine and LTTA Centre, University of Ferrara, Ferrara, Italy; ^2^IRCCS Istituto Ortopedico Galeazzi, Milan, Italy; ^3^Department of Biotechnology and Biosciences, University of Milano-Bicocca, Milan, Italy; ^4^School of Medicine, Universitá Vita-Salute San Raffaele, Milan, Italy

**Keywords:** psoriatic disease, effector memory, central memory, tissue immunosurveillance, skin, T cells

## Abstract

The chemokine receptor CCR4 has emerged as a skin-homing molecule important for the migration of T cells from the blood to the dermis. From our previous data on psoriasis patients, CCR4^+^ memory T cells emerged as a putative recirculating population between skin and blood. Here we focused our attention on the expression of CCR4 and skin-tropic molecules in the different stages of memory T cell differentiation. We analyzed the chemokine receptor profile in CD8^+^ and CD4^+^ CD45RA^−^CCR7^+^ (T_CM_) and CD45RA^−^CCR7^−^ (T_EM_) cells. Subpopulations were further divided on the basis of CD62L expression, and the distribution among the subsets of the skin-homing molecule CLA (Cutaneous Lymphocyte Antigen) was evaluated. The characterization was performed on peripheral blood mononuclear cells isolated from 21 healthy subjects and 24 psoriasis patients. The results indicate that (i) the skin-homing CCR4 marker is mainly expressed in T_CM_ cells, (ii) CCR4^+^ T_CM_ cells also express high level of CLA and that (iii) the more differentiated phenotype T_EM_ expresses CXCR3 and CCR5 but lower level of CCR4 and CLA. This indicates that progressive stages of memory T cell differentiation have profoundly different chemokine receptor patterns, with CD8^+^ T_CM_ displaying a marked skin-tropic phenotype CLA^+^CCR4^+^. Differential skin-tropic phenotype between T_CM_ and T_EM_ cells was observed in both healthy subjects and psoriasis patients. However, patients showed an expanded circulating population of CD8^+^ T_CM_ cells with phenotype CCR4^+^CXCR3^+^ that could play a role in the pathophysiology of psoriasis and possibly in disease recurrence.

## Introduction

Memory T cell subpopulations were first classified on the basis of their phenotype and functional features. The classical definition refers to cells with phenotype CCR7^+^CD45RA^−^ as central memory (T_CM_), CCR7^−^CD45RA^−^ as effector memory (T_EM_) and CCR7^−^CD45RA^+^ as effector T cells (T_EMRA_) (1–3). According to the linear model, these subsets also represent progressive stages of memory T cell differentiation (4, 5). T_CM_ cells have phenotype, functional features and molecular signature intermediate between naïve T cells (CCR7^+^CD45RA^+^) and T_EM_ cells, they express CD62L and home mainly to secondary lymphoid organs. T_EM_ and T_EMRA_, by contrast, are mainly recruited to the inflamed tissues (2, 6–8). T_CM_ cells show high capacity of self-renewal as indicated by high basal and cytokine-induced STAT-5 phosphorylation levels, whereas T_EM_ and T_EMRA_ express higher level of effector molecules and have minimal capacity of self-renewal (9). In recent years, other subsets of memory T cells have entered into the classification. These include memory stem cells (T_SCM_), antigen-experienced circulating T cells with maximal capability of self-renewal and tissue-resident memory T cells (T_RM_), which can persist for long in tissues without exiting into the bloodstream (5). In human skin, they express cutaneous lymphocyte antigen (CLA) and chemokine receptors such as CCR4 and CCR10 (10, 11).

The recent advances in the characterization of tissue resident memory T cells has raised the question of their origin and developmental relationship with the other memory T cell subpopulations. The evidence of a common clonal origin shown for T_CM_ and T_RM_ provided by Gaide et al. now supports the concept that T_CM_ patrolling the tissues can seed antigen-experienced cells that can finally develop into T_RM_ (12–16).

Memory T cells are very heterogeneous in regard to tissue-homing properties and cytokine production. Different chemokine receptor expression profiles have been characterized in association with T cell polarization. According to the current classification, Th1 cells acquire the capacity to produce IFNγ and the expression of chemokine receptors CXCR3, CCR5, and CXCR6, whereas Th2 cells acquire the capacity to produce IL-4 and express receptor CRTh2 and CCR4 (8, 17). Th17 cells have been characterized by the expression of CCR6 as well as CCR4 and Th22 for the expression of both CCR4 and CCR10 (4, 18). Despite these indications, the role of CCR4 as a marker of polarized T cells is still unclear, whereas it is emerging its role in directing T cell migration to the skin.

In a previous study we showed that, in patients with cutaneous psoriasis, CCR4^+^ cells were expanded in the circulating memory (CD45RA^−^) CD4 and CD8 compartments and their percentage positively correlated with the severity of the disease (19).

This has led to the hypothesis that CCR4^+^ T cells may represent a key recirculating population between skin and blood.

This study has the aim to test the hypothesis that skin-tropic chemokine receptors such as CCR4 are differentially distributed among the memory differentiation stages. In addition, we analyzed the skin-tropic phenotype of patients diagnosed with cutaneous psoriasis.

## Materials and Methods

### Study Design

The study had as a first aim the characterization of the chemokine expression profile in memory T cell subpopulations T_CM_ and T_EM_. Specifically, CCR4, CXCR3, CCR5, and CCR6 were analyzed on CD8^+^ and CD4^+^ CD45RA^−^ CCR7^+^ (T_CM_) and CD45RA^−^CCR7^−^ (T_EM_) cells. In order to define the skin-tropic phenotype within the memory T cell subpopulations, we analyzed the distribution of the skin-homing molecule CLA among the subsets of memory T cells. The characterization was performed on peripheral blood mononuclear cells (PBMCs) isolated from 21 healthy subjects and then compared with a group of 24 psoriasis patients.

In some experiments T_CM_ and T_EM_ cells were further divided on the basis on CD62L expression. CCR4 and CLA were evaluated on CCR7^+^CD62L^+^CD45RA^−^ and CCR7^+^CD62L^−^CD45RA^−^ cells.

### Human Subjects and Patients Recruitment

Healthy control subjects with negative family and personal anamnesis for psoriasis as well as patients with psoriasis were recruited by the Department of Dermatology, Istituto di Ricovero e Cura a Carattere Scientifico Istituto Ortopedico Galeazzi (Milan, Italy) within the clinical study approved by the local Ethical Committee (Comitato Etico dell'Ospedale San Raffaele, Milan, Italy) and registered on ClinicalTrials.gov (NCT03374527).

Subjects undergoing treatment with cyclosporin A, methotrexate, systemic corticosteroids or any other immunosuppressant or biotechnological agents within at least 3 weeks prior to the collection of blood samples were excluded from the study. Systemic autoimmune diseases such as type 1 diabetes, neoplastic diseases, chronic or acute infections were used as exclusion criteria. Some demographic and clinical characteristics are summarized in Supplemental Table 1.

Peripheral venous blood samples were collected from each patient and healthy subject into BD Vacutainer tubes (BD Biosciences, Franklin Lakes, NJ, USA) containing EDTA for flow cytometry analysis.

### T Cell Isolation and FACS Analysis

Peripheral blood mononuclear cells (PBMCs) were prepared from whole blood from healthy subjects and patients by Ficoll gradient centrifugation (Lympholyte®, Cederlane® Hornby, Ontario, Canada) as previously described (20). For the phenotypic characterization, unstimulated PBMCs were stained with different combinations of fluorochrome-conjugated antibodies. We used combinations of fluorochrome-conjugated antibodies against: CD4 (RPA-T4), CD8 (SK1), CD45RA (HI100), CCR4 (1G1), CXCR3 (1C6), CCR5 (2D7/CCR5), CCR6 (11A9) (all from BD Biosciences) and CD3 (REA613), CCR7 (REA546), CD62L (145/15) and the skin-tropic CLA molecule (REA1101) (all from Miltenyi Biotec GmbH). To automatically assess fluorescence compensation, MACS Comp Bead Kits (Miltenyi Biotec) as well as the fluorochrome-conjugated antibodies were used. To evaluate non-specific fluorescence when defining positive events, we used Fluorescence Minus One (FMO) controls which contains the multicolor staining combination except the antibody in the detector of interest (Supplemental Figures 1, 2) (21–23). As additional control, samples were stained with isotype control antibodies to confirm the threshold for the marker of interest.

Samples were acquired using FACSAriaII (BD biosciences) flow cytometer (19, 24) and analyzed with the FlowJo software (Tree Star, Ashland, OR). A representative gating strategy is shown in Supplemental Figure 3.

### Statistical Analysis

The Gaussian distribution of overall data was evaluated using the Shapiro–Wilk test. Statistical comparisons between each subpopulation in individual subjects was performed by paired or unpaired analysis calculated with non-parametric tests (Wilcoxon signed-rank test or Mann–Whitney non-parametric *U*-test, as appropriated) when no Gaussian distribution was found, otherwise Student's *t*-test was used. Statistical analysis was performed using GraphPad Prism 6 software.

## Results

### Differential Expression of CCR4 in CD8^+^ T_CM_ and T_EM_ Cells

From our previous data on psoriasis patients, CCR4^+^ memory T cells emerged as a putative recirculating population between skin and blood. Here we focused our attention on the expression of CCR4 in different subsets of memory T cells.

We analyzed the chemokine receptor expression in circulating CD8^+^ and CD4^+^ memory T cells with CD45RA^−^CCR7^+^ (T_CM_) and CD45RA^−^CCR7^−^ (T_EM_) phenotype in 21 healthy subjects. Specifically, we evaluated the expression of chemokine receptors CCR4, CCR6, CXCR3 and CCR5.

**Figure 1:**
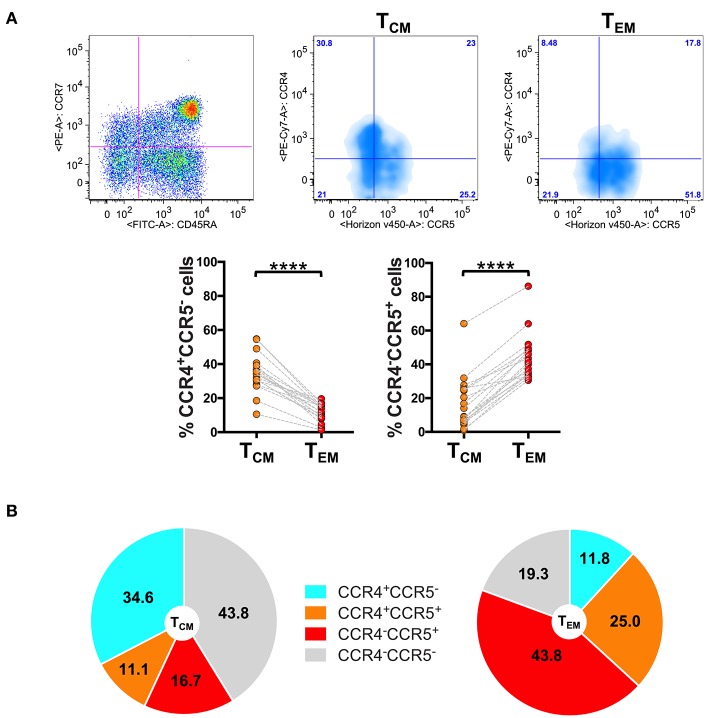
Differential expression of CCR4 in CD8^+^ T_CM_ and T_EM_ cells. PBMCs isolated from healthy control subjects were stained for CD8, memory T cell phenotype markers (CD45RA and CCR7) and for chemokine receptors CCR4 and CCR5. **(A)** CD8^+^ T cells gated as CD45RA^−^CCR7^+^ T_CM_ and CD45RA^−^CCR7^−^ T_EM_ were analyzed for the expression of CCR4 and CCR5. Representative analysis is shown in the figure. The axis scales for fluorescence are reported as log. Statistical analysis of the differences was performed by Mann–Whitney test. *p*-values < 0.05 were considered significant: ****p < 0.0001. **(B)** Mean values of the percentage of CCR4/CCR5 subpopulations among T_CM_ and T_EM_ cells were shown in pie charts.

The results of the analysis revealed that the skin-homing CCR4 receptor was expressed at markedly higher level in T_CM_ than in T_EM_ cells. Indeed, as shown in Figure 1A, in CD8^+^ T cells the percentage of CCR4^+^CCR5^−^ cells significantly decreased from T_CM_ to T_EM_ cells (*p* < 0.0001). By contrast, CCR5^+^CCR4^−^ cells that were present at low frequency in T_CM_ strongly augmented in the T_EM_ compartment.

Representing the chemokine receptor profiles in the different subsets of memory T cells (Figure 1B), we evidenced that CD8^+^ T_CM_ cells contained a high percentage of CCR4^+^CCR5^−^ cells (34.6 ± 11.0%; mean ± SD) whereas they contained 16.7 ± 15.2% of cells with phenotype CCR5^+^CCR4^−^. The chemokine receptor profile dramatically changed in T_EM_ cells where the percentage of CCR4^+^CCR5^−^ cells lowered down to 11.8 ± 5.2% whereas the percentage of CCR5^+^CCR4^−^ cells increased to 43.8 ± 13.7% in the T_EM_ compartment.

These results led to the hypothesis that CCR4 could represent a specific feature of CD8^+^ T cells with central memory phenotype.

**Figure 2:**
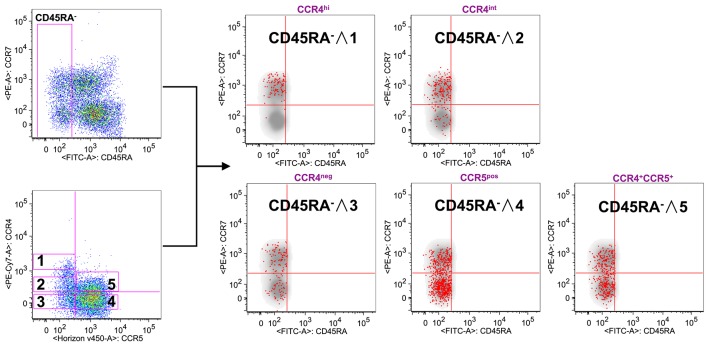
CCR4 expression characterizes the T_CM_ compartment. PBMCs isolated from healthy control subjects were stained for CD8, memory T cell phenotype markers (CD45RA and CCR7) and for chemokine receptors CCR4 and CCR5. **(A)** CD8^+^ T cells were analyzed for the memory phenotype according to CD45RA, CCR7 expression and for the expression of the chemokine receptors CCR4 and CCR5. On the basis of the chemokine receptor expression we identified five subsets CCR4^hi^ (gate 1), CCR4^int^ (gate 2), CCR4^−^CCR5^−^ (gate 3), CCR4^−^CCR5^+^ (gate 4) and CCR4^+^CCR5^+^ (gate 5). These five subsets were superimposed to the density plot of the CD45RA^−^ gated cells. Each red dot identifies cells from the corresponding subset as reported in the figure. The axis scales for fluorescence are reported as log.

To verify this possibility, we used the reverse approach (Figure 2). CD8^+^ gated T cells were analyzed on the basis of CCR7 and CD45RA expression or for the expression of CCR4 and CCR5. Total CD8^+^ gated cells were divided into five subpopulations: CCR4 highly expressing cells (CCR4^hi^), cells expressing intermediate level of CCR4 (CCR4^int^), CCR4 and CCR5 double negative cells (CCR4^neg^), cells expressing CCR5 (CCR5^pos^) and cells co-expressing CCR4 and CCR5 (CCR4^+^CCR5^+^). Overlay analysis of these selected areas with CD45RA^−^ CD8^+^ T cells showed that CCR4^hi^CCR5^−^ cells were almost entirely central memory (*p* < 0.0001, Supplemental Table 2).

CCR4^int^ CD8^+^ T cells had a trend toward an accumulation in the T_CM_ population whereas the CCR4^−^CCR5^+^ cells, though being detectable in all the selected CD45RA^−^ populations, were for the vast majority in the T_EM_ compartment (*p* < 0.0001, Supplemental Table 2).

**Figure 3:**
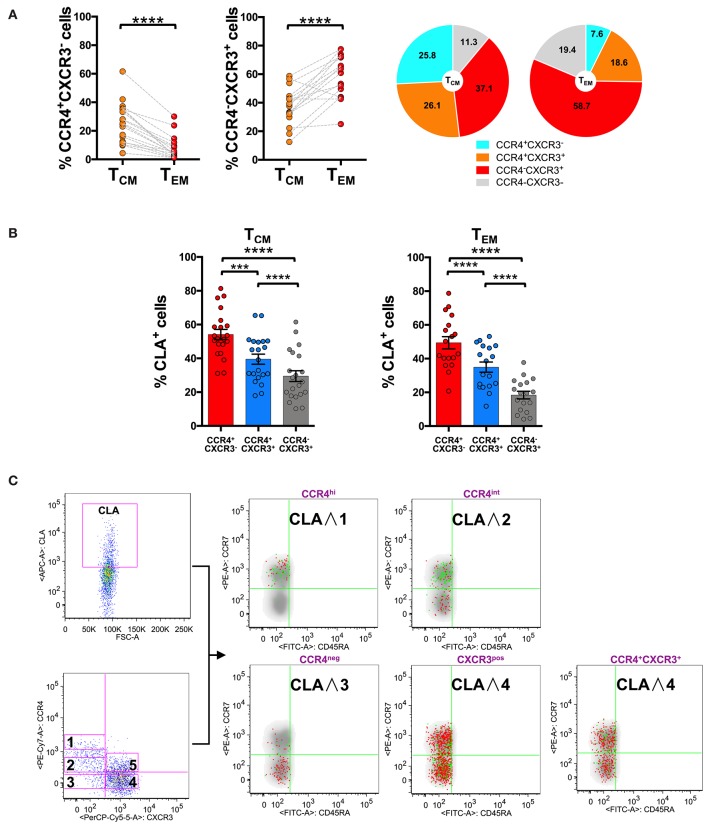
CLA expression is maximal in CCR4^+^ cells and considerably decreases in CXCR3^+^ cells. PBMCs isolated from healthy control subjects were stained for CD8, memory T cell phenotype markers (CD45RA and CCR7), for chemokine receptors CCR4, CXCR3, and for CLA. **(A)** CD8^+^ T cells gated as T_CM_ and T_EM_ were analyzed for the expression of CCR4 and CXCR3. Statistical analysis of the differences was performed by Mann-Whitney test. *p*-values < 0.05 were considered significant: ***p < 0.001, ****p < 0.0001. Mean values for CCR4/CXCR3 subpopulations among T_CM_ and T_EM_ cells were shown in pie charts. **(B)** T_CM_ and T_EM_ cells were analyzed for differences in the percentage of CLA^+^ cells in the CCR4^+^CXCR3^−^, CCR4^+^CXCR3^+^, and CXCR3^+^ subpopulations using Mann-Whitney test. *p*-values < 0.05 were considered significant: ****p < 0.0001. **(C)** Reverse analysis is shown in the panel. Total memory CD45RA^−^ CD8^+^ T cells were gated and analyzed for the expression of CLA and for chemokine receptors CCR4/CXCR3. On the basis of the chemokine receptor expression we identified five subsets CCR4^hi^ (gate 1), CCR4^int^ (gate 2), CCR4^−^CXCR3^−^ (gate 3), CCR4^−^CXCR3^+^ (gate 4), CCR4^+^CXCR3^+^ (gate 5). The five subsets of CCR4/CXCR3 expressing cells were overlaid to the density plot of CD45RA^−^ cells. Each red dot represents a cell from the corresponding subset. Among those dots, green dots identify cells of the gated subsets (CCR4^hi^, CCR4^int^, CCR4^−^CXCR3^−^, CCR4^−^CXCR3^+^, and CCR4^+^CXCR3^+^) that co-express CLA. The axis scales for fluorescence are reported as log.

Analysis of CCR4 and CXCR3 expression and distribution in CD8^+^ T_CM_ and T_EM_ compartments evidenced that CCR4^+^CXCR3^−^ cells also accumulated in T_CM_ (25.8 ± 14.0% in T_CM_ vs. 7.6 ± 7.9% in T_EM_). CXCR3^+^CCR4^−^ cells conversely represented the 37.1 ± 12.0% of T_CM_ cells and increased to 58.7 ± 14.3% in the T_EM_ compartment (Figure 3A).

Interestingly, a considerable fraction of cells in the T_CM_ compartment showed a double positive phenotype (CCR4^+^CXCR3^+^ 26.1 ± 12.7%), that was reduced in the T_EM_ compartment (18.6 ± 10.0%, *p* < 0.05).

These data evidenced a selective accumulation of the skin-tropic CCR4 chemokine receptor in T_CM_ cells and a clear shift toward a CXCR3^+^ and CCR5^+^ phenotype in the T_EM_ compartment.

Similar results were obtained in CD4^+^ T cells (Supplemental Figure 4). In this case the percentage of CCR4^+^CCR5^−^ was 39.9 ± 9.1% in the T_CM_ and was lowered to 26.0 ± 7.5% in the T_EM_ compartment. Conversely, CCR5^+^CCR4^−^ cells that were minimally represented in T_CM_ (4.7 ± 3.3%) increased to 27.6 ± 11.8% in T_EM_ cells. Analyzing the CCR4^+^CXCR3^−^ subset, a significant difference was still observed between T_CM_ (35.8 ± 7.7%) and T_EM_ (29.9 ± 8.7%) cells even if the difference was less pronounced (Supplemental Figure 5).

The analysis of the distribution of CCR6^+^CXCR3^−^, CCR6^+^CXCR3^+^, and CXCR3^+^CCR6^−^ in the two compartments showed that CCR6^+^CXCR3^−^ cells were represented with similar frequencies in the two subsets of memory T cells (Supplemental Figure 6).

To confirm this first part of the results, we searched for gene expression data in individual immune cell populations using ImmGen Browser (http://www.immgen.org/databrowser/index.html) (25). Dataset analysis confirmed that CCR4 gene is expressed at markedly higher level in the T_CM_ than in T_EM_ subset whereas CCR5 gene is expressed by T_EM_ cells and at markedly lower level by T_CM_ (Supplemental Figure 7).

These data finally confirm that the subsets of memory T cells have markedly different patterns of chemokine receptor expression.

### CLA Expression Is Maximal in CCR4^+^ Subset and Considerably Decreases in CXCR3^+^ Subset

To investigate further the skin-tropic phenotype, we analyzed CLA expression in different memory T cell differentiation stages. As shown in Figure 3B, in the CD8 compartment, the expression of the skin-homing molecule CLA was maximal in CCR4^+^ T cells and progressively decreases toward the CXCR3^+^ phenotype.

Analyzing the localization of the CLA^+^ fraction among the CD8^+^ memory T cells together with the CCR4^high^, CCR4^int^, and CXCR3 expression using the reverse approach, we found that CLA co-localized with CCR4 (green dots) mainly in the T_CM_ cells (Figure 3C). Indeed CCR4^hi^CXCR3^−^ cells co-express CLA mainly in the T_CM_ compartment (*p* = 0.0017, Supplemental Table 2) whereas in CCR4^−^CXCR3^+^ cells CLA expression was more abundant in the effector memory subset (*p* < 0.0001, Supplemental Table 2).

Accumulation of CLA^+^ cells in CCR4^+^ subsets was observed also in the total memory CD4 compartment, however, CLA expression was not significantly different in CCR4^+^ T_CM_ and T_EM_ subsets (data not shown). This difference underlines that skin-tropic features of T_CM_ cells are more evident in the CD8 compartment.

### CD62L^+^ CCR7^+^ Central Memory T Cells Selectively Express a Skin-Tropic Phenotype

**Figure 4:**
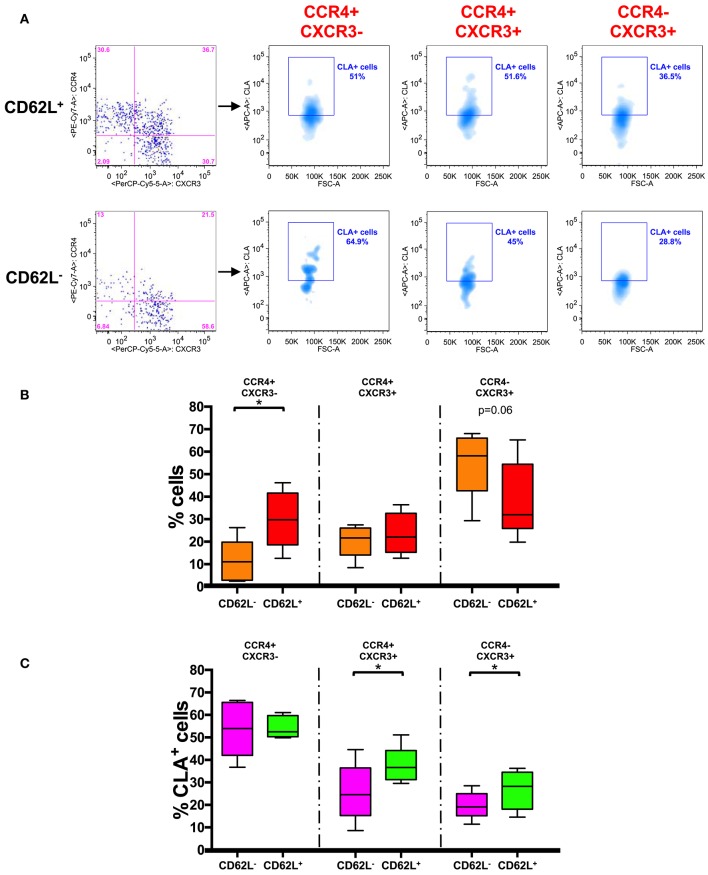
CD62L^+^ CCR7^+^ central memory T cells have a skin-tropic phenotype. **(A)** Representative analysis of chemokine receptor expression in CD62L^+/−^ gated CD45RA^−^ CCR7^+^ (T_CM_) CD8^+^ T cells. Each subset (CCR4^+^CXCR3^−^, CCR4^+^CXCR3^+^, and CCR4^−^CXCR3^+^) in CD62L^+^ and CD62L^−^ gates was also analyzed for the expression of CLA. The axis scales for fluorescence are reported as log and the axis scale for FSC is reported as linear. **(B)** CD45RA^−^CCR7^+^ (T_CM_) CD8^+^ T cells were gated on the basis of the expression of CD62L and analyzed for the expression of chemokine receptors (CCR4/CXCR3). The percentage of positive cells for each phenotype in CD62L^+^ and CD62L^−^ gated cells was represented as Tukey's boxplot. Significance of the differences was calculated using Student's *t*-test for paired samples. *p*-values < 0.05 were considered significant: *p < 0.05. **(C)** CD45RA^−^ CCR7^+^ (TCM) CD8^+^ T cells were gated on the basis of the expression of CD62L and further divided into different chemokine expressing subsets. The percentages of CLA^+^ cells in the different subsets are represented as Tukey's boxplot. Significance of the differences was calculated using Student's *t*-test for paired samples. *p*-values < 0.05 were considered significant: *p < 0.05.

In a recent study two distinct populations of recirculating memory T cells have been described within the CCR7^+^ memory T cell compartment and were distinguished on the basis of the expression of CD62L (26). We therefore analyzed the frequency of CCR4^+^ cells in CD62L^+^ and CD62L^−^ fractions of CD45RA^−^CCR7^+^ cells (Figure 4A).

We found that CCR4^+^ cells were actually present at significantly higher percentage in CD62L^+^ T_CM_ fraction whereas CXCR3^+^ cells accumulated mainly in the CD62L^−^ T_CM_ compartment (Figure 4B). Notably CLA was found to be expressed at a higher level in the CD62L^+^ fraction of T_CM_ cells (40.40 ± 1.6%) compared to CD62L^−^ (22.64 ± 3.1%) (*p* < 0.001, data not shown). A significantly higher percentage of CLA^+^ cells was observed in CCR4^+^CXCR3^+^ and CCR4^−^CXCR3^+^ subsets of CD62L^+^ T_CM_ cells compared to the same subsets of CD62L^−^ T_CM_ cells. No differences were observed in CCR4^+^CXCR3^−^ cells where CLA was expressed at high level in both CD62L^+^ and CD62L^−^ T_CM_ subsets (Figure 4C).

This finding further restricts the subset of skin-tropic T cells within the CD8^+^ T_CM_ compartment and defines CLA^+^CCR4^+^CD62L^+^ CD8^+^ T_CM_ cells as a highly skin-tropic population.

### Skin-Tropic Phenotype of Circulating CD8^+^ T Cells in Psoriasis Patients

To explore the possibility of a role of the skin-tropic subset of CD8^+^ T_CM_ cells in skin immunopathology we performed a comparison of chemokine receptor expression in T_CM_ and T_EM_ compartments of psoriasis patients and we compared the frequency of T_CM_ and T_EM_ chemochine receptor positive cells between patients and healthy subjects.

**Figure 5:**
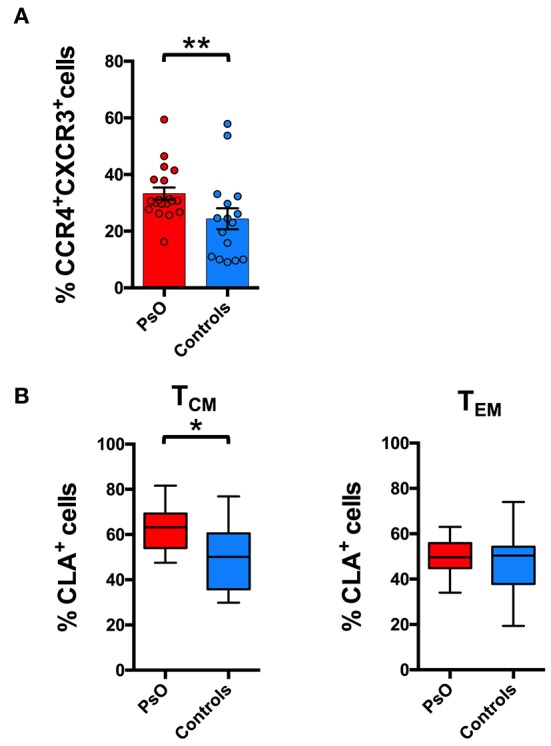
Skin-tropic phenotype of circulating CD8^+^ T cells in psoriasis patients. **(A)** CD8^+^ cells from patients with cutaneous psoriasis (PsO) and healthy subjects were analyzed for the percentage of cells expressing chemokine receptors CCR4/CXCR3 in the CD45RA^−^CCR7^+^ (T_CM_) compartment. Statistical analysis of the differences was performed by Mann–Whitney test. *p*-values < 0.05 were considered significant: **p < 0.01. **(B)** CD8^+^ cells gated on the basis of the memory T_CM_ or effector T_EM_ phenotype, from patients with cutaneous psoriasis (PsO) and healthy subjects were analyzed for the percentage of CLA expressing cells in the CCR4^+^CXCR3^−^ subpopulation. Statistical analysis of the differences was performed by Mann–Whitney test. *p*-values <0.05 were considered significant: *p < 0.05.

In the psoriasis patient cohort there was the same difference in the chemokine receptor percentage between T_CM_ and T_EM_ cells as observed in healthy subjects (Supplemental Figure 8). However, the comparison of the phenotype of circulating CD8^+^ T cells between psoriasis patients and healthy subjects evidenced a significant increase in the percentage of CCR4^+^CXCR3^+^ CD8^+^ T_CM_ cells in the circulation of psoriasis patients (Figure 5A). In these patients we also evidenced a significantly higher CLA expression in the CD8^+^ CCR4^+^CXCR3^−^ T_CM_ but not in the T_EM_ compartment (Figure 5B). Correlation analysis with the PASI (Psoriasis Area and Severity Index) score indicates that the circulating levels of these subsets did not directly correlate with the severity of the cutaneous disease (data not show). Nevertheless, they could be involved in recirculating events associated with disease recurrence or systemic manifestations.

## Discussion

From the results of this study it emerged that the skin-homing CCR4 marker is mainly expressed in T_CM_ cells. CCR4^+^ T_CM_ cells also express high level of CLA whereas the more differentiated phenotype T_EM_ expresses CXCR3 and CCR5 but lower level of CCR4 and CLA.

This indicates that progressive stages of memory T cell differentiation have different chemokine receptor profiles, with T_CM_ displaying a marked skin-tropic phenotype CLA^+^CCR4^+^.

It is important to note that skin-tropic features are progressively lost in more advanced stages of memory T cell differentiation and in CXCR3^+^ phenotype.

The evidence of a selective accumulation of the skin-tropic CCR4 chemokine receptor in the central memory compartment suggests a preferential migration of the small population of CCR7^+^ CD8^+^ memory T cells toward the skin. By contrast expression of CXCR3 and CCR5 receptors for the inflammatory chemokines CXCL10 and CCL5 is clearly confirmed as a feature of cells in a more advanced stage of differentiation (8, 27). This underlines a clear distinction between T_CM_ and T_EM_ migratory properties enlightening skin-tropic features for T_CM_ cells and migratory properties toward inflamed tissues for T_EM_ cells.

A prevalence of the CCR4^+^ phenotype in T_CM_ cells has been occasionally reported in previous works (28, 29), however more controversial findings have been reported in recent studies thus not strengthening this evidence (4).

Our results strongly reinforce the evidence of the first reports and enlighten a possible new classification of chemokine receptor and skin-tropic molecule expression as a function of memory T cell differentiation.

CCR4 is important for the mechanism by which T cells migrate into the dermis from the blood and has been defined as a skin-homing receptor that is up-regulated by memory T cells primed in skin-draining lymph nodes (2, 30). To enter non-inflamed skin, these T cells must interact with the low constitutive levels of homing molecules expressed on resting endothelium such as CLA ligand E-selectin and the CCR4 ligand CCL17 (31).

Under physiological conditions CCR4 has been reported as a non-redundant, necessary component of skin-specific lymphocyte trafficking (32). Its role could however change under inflammatory conditions, where other chemokines could play a major role in the recruitment of effector memory/effector T cells (27, 33). Consistent with this view, different groups evidenced that T cell mediated skin-inflammation is largely independent of CCR4 and rather requires CXCR3 (34, 35). Along this line, in psoriatic patients both gene expression analysis and circulating T cell phenotype suggest recruitment of CXCR3^+^ and CCR5^+^ cells to the inflamed skin associated with upregulation of the skin expression of their ligand CXCL10 and CCL5 (19, 24).

CCR4 could therefore play a major role in skin patrolling by central memory T cells rather than in mediating the recruitment of effectors cells to the inflamed skin. Consistently, in a recent study it has emerged that T_CM_ cells play a role in tissue immunosurveillance. In line with this concept, the skin compartment had been previously suggested as an important site for lymphocyte differentiation and antigen encounter and was proposed for the definition of “peripheral lymphoid organ” (15, 36).

Our results strengthen the role of the skin as a preferential trafficking site for T_CM_ cells, where it is possible that antigen encounter occurs. It also defines the limited subpopulation of circulating CD8^+^ T_CM_ cells as a subset with high skin-tropic features. These cells after antigen encounter and under appropriate environmental conditions could possibly give rise to non-circulating T_RM_ cells. In immunopathological skin conditions such as psoriasis, the expanded subset of T_CM_ cells expressing CCR4 and CXCR3 could play a role in disease recurrence or redistribution to distant sites such as joint synovial tissues and enthesis.

Our data therefore add a new evidence to the concept of physiological skin trafficking and immunosurveillance, focusing the attention on a specific subpopulation of cells with central memory and CCR4^+^ phenotype. It also opens the question of the physiological role of this phenomenon and its possible alterations in pathological conditions.

## Data Availability Statement

The datasets generated for this study are available on request to the corresponding author.

## Ethics Statement

The study was approved by the local Ethical Committee (Comitato Etico dell'Ospedale San Raffaele, Milan, Italy) (30IOG 17/07/2014), and written informed consent was obtained from all patients and healthy subjects before they entered the study, which was performed in accordance with the Declaration of Helsinki. The study was registered on ClinicalTrials.gov, Identifier: NCT03374527.

## Author Contributions

FC performed the flow cytometry experiments, analyzed the data, prepared the figures, and contributed to the writing of the manuscript. MD and AA selected the patients and control subjects to be recruited in the study, collected samples, and clinical data. FG participated in the supervision of the research activities, and in data interpretation. PS participated in data interpretation and contributed to the final version of the manuscript. GB participated in the coordination of the activities between clinical and research groups. ER designed the study, coordinated the research activities, and wrote the final version of the manuscript.

### Conflict of Interest

The authors declare that the research was conducted in the absence of any commercial or financial relationships that could be construed as a potential conflict of interest.
