# Supplementary material for: CCR4+ Skin-Tropic Phenotype as a Feature of Central Memory CD8+ T Cells in Healthy Subjects and Psoriasis Patients
Source: Front Immunol. 2020 Apr 3;11:529. doi: 10.3389/fimmu.2020.00529 (PMC7147166; doi:10.3389/fimmu.2020.00529)
Supplement: Supplementary file 1 [file Data_Sheet_1.pdf]

CCR4$^+$ skin-tropic phenotype as a feature of central memory CD8$^+$ T cells in healthy subjects and psoriasis patients.

Fabio Casciano[1], Marco Diani[2], Andrea Altomare[2], Francesca Granucci[3], Paola Secchiero[1], Giuseppe Banfi[2,4], and Eva Reali[2*]

[1]Department of Morphology, Surgery and Experimental Medicine and LTTA Centre, University of Ferrara, Ferrara, Italy.

[2]IRCCS Istituto Ortopedico Galeazzi, Milan, Italy.

[3]Department of Biotechnology and Biosciences, University of Milano-Bicocca, Milan, Italy.

[4]Università Vita-Salute San Raffaele, Milan, Italy.

* Corresponding author: Correspondence and requests for materials should be addressed to E.R. (email: eva.reali@grupposandonato.it).

Supplementary Information

Cohort	N	M	F	Age	PASI
PsO	24	18	6	45±13	12.2±4.8
Controls	21	11	10	40±11	NA

Age, PASI presented as mean ± SD.

Supplemental Table 1. Demographic Characteristics of Patients and Controls

Cell population (fraction)	T_{CM}	T_{EM}	p value
$CCR4^{hi}CCR5^{-}$ (1)	**77.01±17.00**	22.86±17.03	<0.0001
$CCR4^{int}CCR5^{-}$ (2)	**55.56±26.00**	44.31±26.02	0.4017
$CCR4^{-}CCR5^{+}$ (4)	15.63±13.34	**84.25±13.39**	<0.0001
$CCR4^{+}CCR5^{+}$ (5)	25.93±20.50	**73.95±20.60**	0.0004
$CCR4^{hi}CXCR3^{-}$ (1)	**74.39±14.38**	25.51±14.39	<0.0001
$CCR4^{-}CXCR3^{-}$ (3)	17.25±10.81	**82.63±10.67**	<0.0001
$CCR4^{-}CXCR3^{+}$ (4)	29.38±14.19	**70.51±14.25**	<0.0001
$CCR4^{+}CXCR3^{+}$ (5)	39.32±19.83	**60.57±19.91**	0.0376
$CLA^{+}CCR4^{hi}CXCR3^{-}$ (1)	**66.46±20.20**	33.30±20.15	0.0017
$CLA^{+}CCR4^{-}CXCR3^{-}$ (3)	31.21±18.51	**68.81±18.13**	0.0002
$CLA^{+}CCR4^{-}CXCR3^{+}$ (4)	36.23±8.61	**63.54±8.57**	<0.0001

Supplemental Table 2. Analysis of T_{CM} and T_{EM} cells in chemokines receptors expressing subsets

The percentage of each fraction, reported in Figure 2 and Figure 3C, in T_{CM} and T_{EM} cells is shown in the table (n=10). p values were calculated using Unpaired t-test.

SUPPLEMENTAL FIGURES

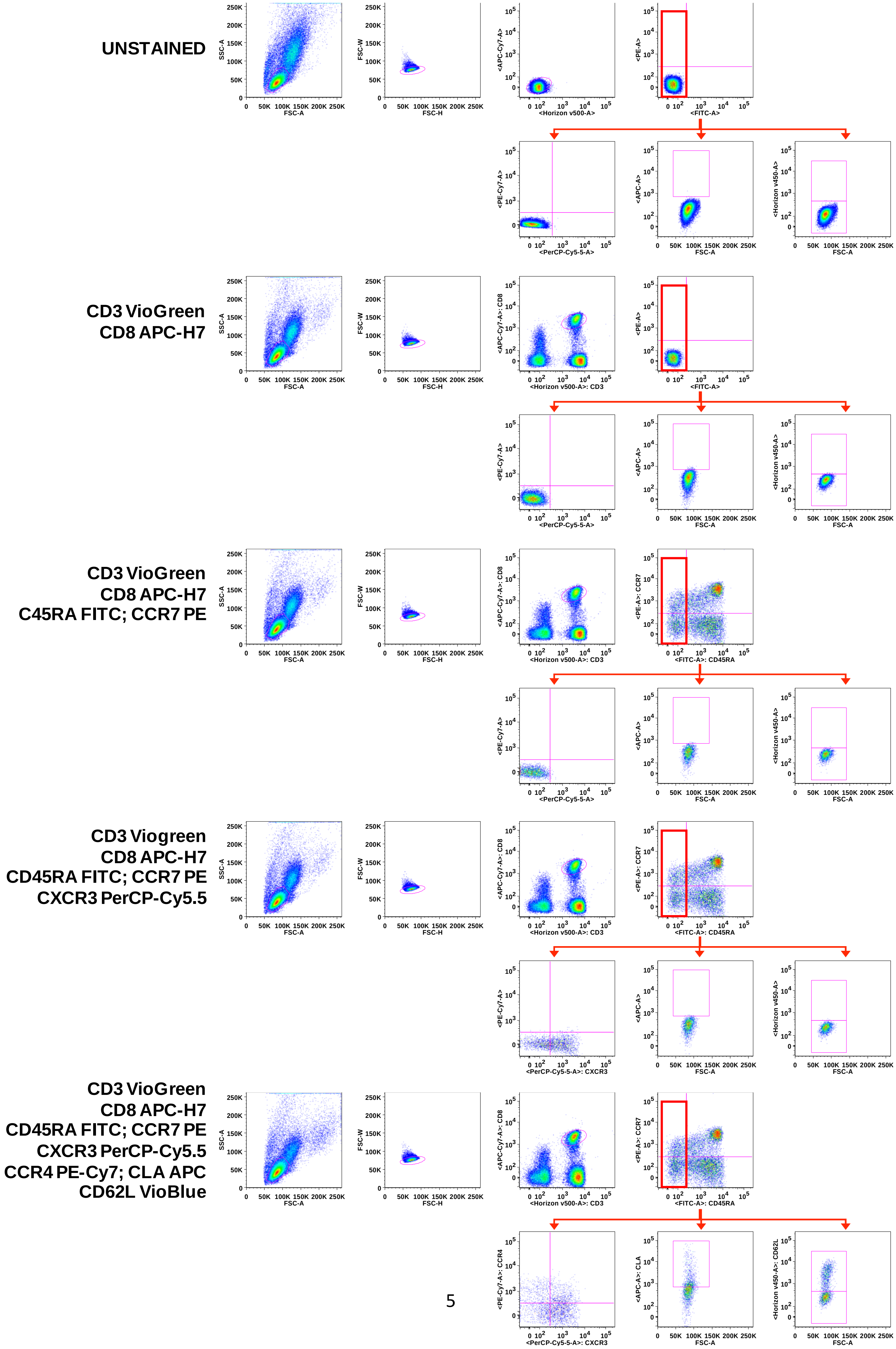
UNSTAINED
CD3 VioGreen
CD8 APC-H7
CD3 VioGreen
CD8 APC-H7
C45RA FITC; CCR7 PE
CD3 Viogreen
CD8 APC-H7
CD45RA FITC; CCR7 PE
CXCR3 PerCP-Cy5.5
CD3 VioGreen
CD8 APC-H7
CD45RA FITC; CCR7 PE
CXCR3 PerCP-Cy5.5
CCR4 PE-Cy7; CLA APC
CD62L VioBlue
SSC-A
FSC-A
FSC-W
FSC-H
<APC-Cy7-A>
<Horizon v500-A>
<PE-A>
<FITC-A>
<PE-Cy7-A>
<PerCP-Cy5-5-A>
<APC-A>
<Horizon v450-A>
<APC-Cy7-A>: CD8
<Horizon v500-A>: CD3
<PE-A>: CCR7
<FITC-A>: CD45RA
<PerCP-Cy5-5-A>: CXCR3
<PE-Cy7-A>: CCR4
<APC-A>: CLA
<Horizon v450-A>: CD62L

Supplemental figure 1

PBMCs isolated from healthy donors were analyzed by flow cytometry.

Example of Fluorescence Minus One (FMO) staining. The axis scales for fluorescence are reported as log, the axis scales for SSC, FSC are reported as linear.

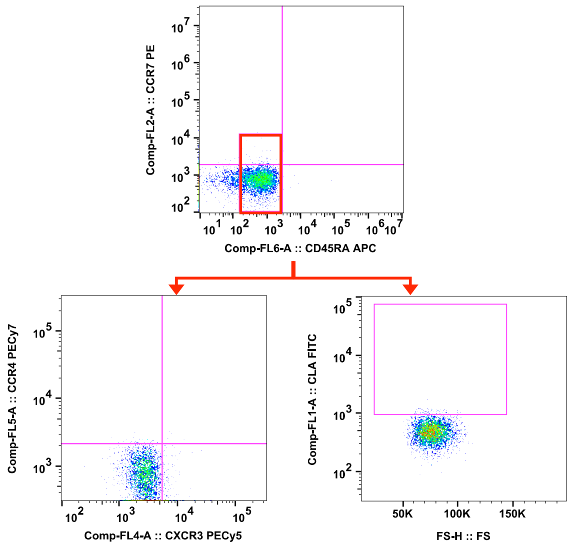

Supplemental figure 2

PBMCs isolated from healthy donors were analyzed by flow cytometry.

Example of Isotype Control Staining. The axis scales for fluorescence are reported as log, the axis scales for FSC are reported as linear.

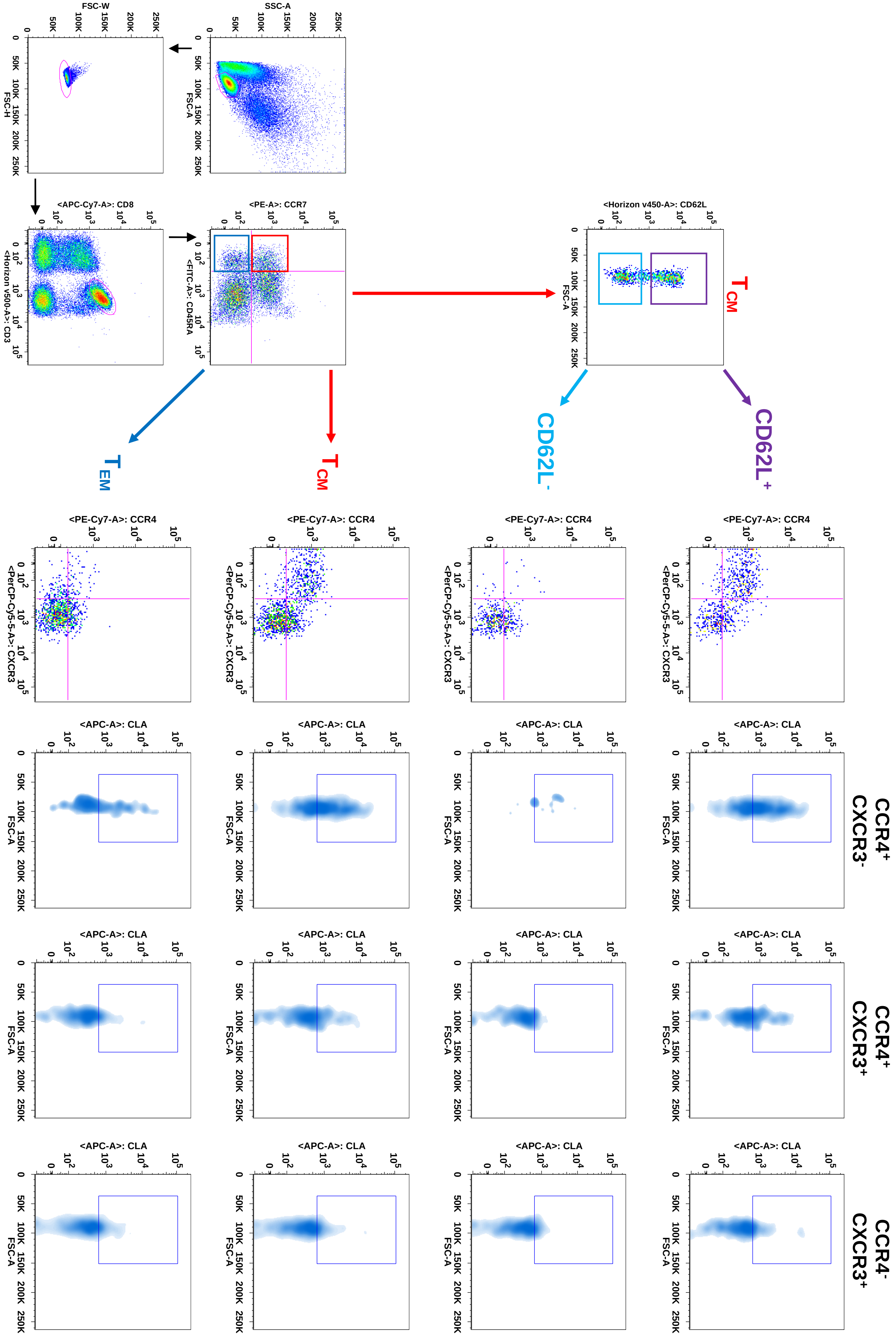
SSC-A
FSC-A
FSC-W
FSC-H
<APC-Cy7-A>: CD8
<Horizon v500-A>: CD3
<PE-A>: CCR7
<FITC-A>: CD45RA
<Horizon v450-A>: CD62L
T CM
CD62L+
CD62L-
T EM
<PE-Cy7-A>: CCR4
<PerCP-Cy5-5-A>: CXCR3
<APC-A>: CLA
CCR4+ CXCR3-
CCR4+ CXCR3+
CCR4- CXCR3+

Supplemental figure 3

PBMCs isolated from healthy donors were analyzed by flow cytometry.

Example of gating strategy. The axis scales for fluorescence are reported as log, the axis scales for SSC, FSC are reported as linear.

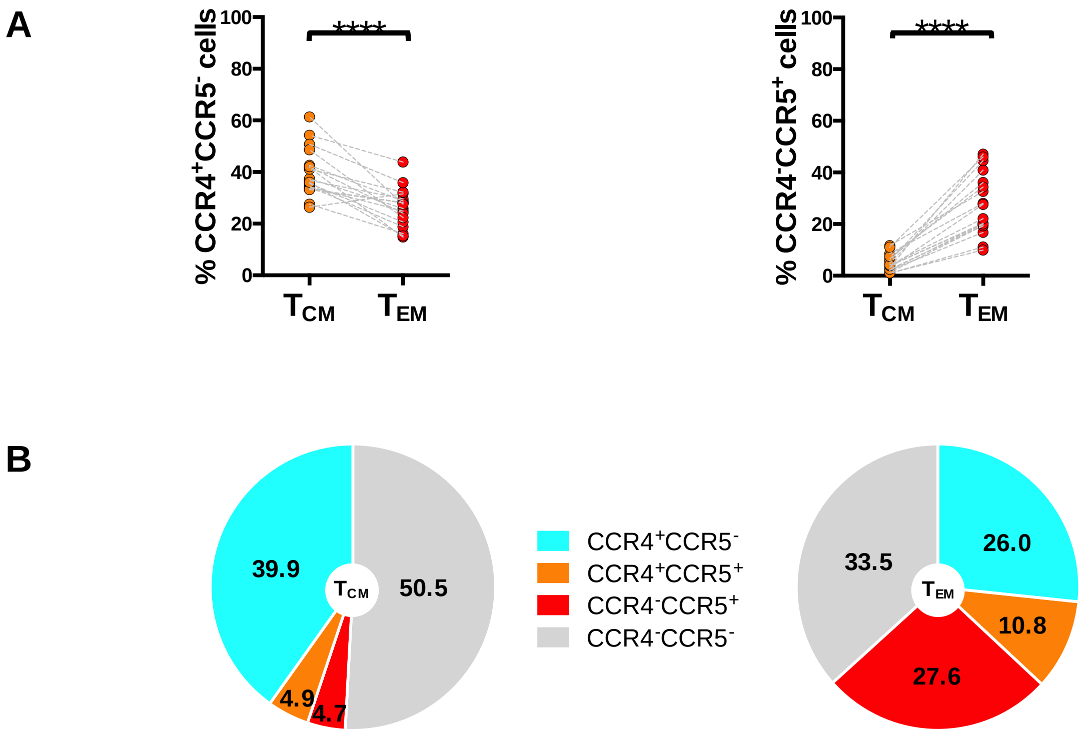

Supplemental figure 4

PBMCs isolated from healthy donors were analyzed by flow cytometry.

(A) $CD4^+$ T cells gated as $CD45RA^+CCR7^+$ T_{CM} and $CD45RA^+CCR7^-$ T_{EM} were analyzed for the expression of CCR4 and CCR5.

Statistical analysis of the differences was performed by Mann-Whitney test. p values <0.05 were considered significant: **** p<0.0001.

(B) Mean values of the percentages of CCR4/CCR5 subpopulations among $CD4^+$ T_{CM} and T_{EM} were shown in pie charts.

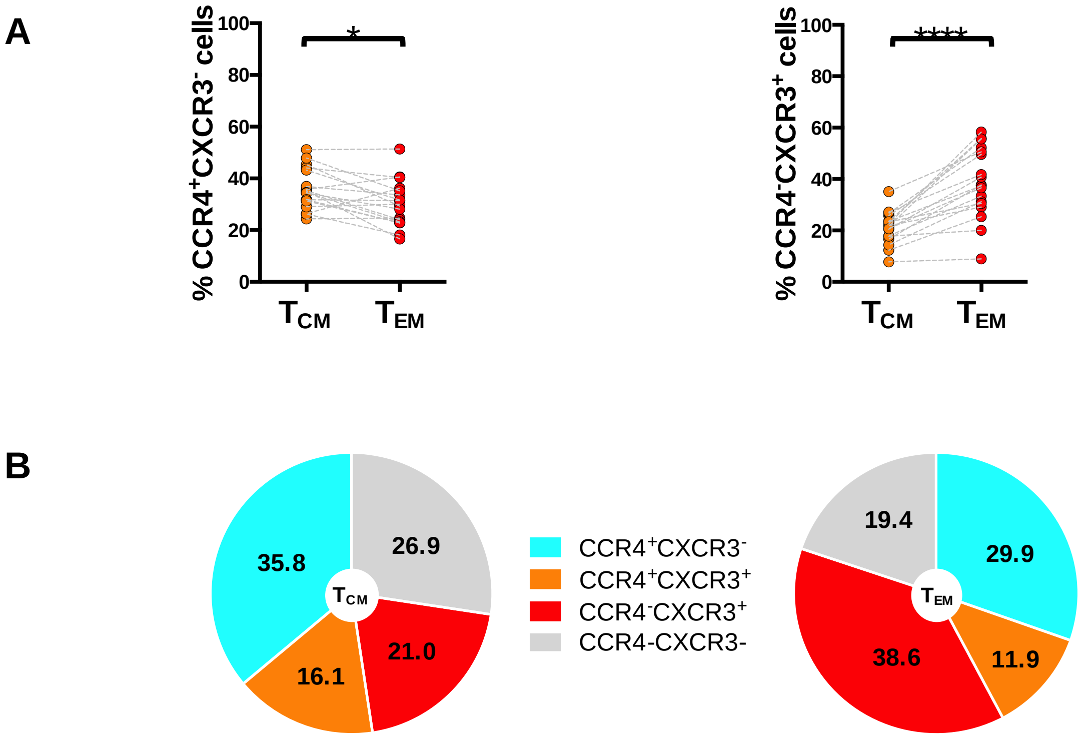

Supplemental figure 5

PBMCs isolated from healthy donors were analyzed by flow cytometry.

(A) $CD4^+$ T cells gated as $CD45RA^+CCR7^+$ T_{CM} and $CD45RA^+CCR7^-$ T_{EM} were analyzed for the expression of CCR4 and CXCR3.

Statistical analysis of the differences was performed by Mann-Whitney test. p values <0.05 were considered significant: * p<0.05, **** p<0.0001.

(B) Mean values of the percentages of CCR4/CXCR3 subpopulations among $CD4^+$ T_{CM} and T_{EM} were shown in pie charts.

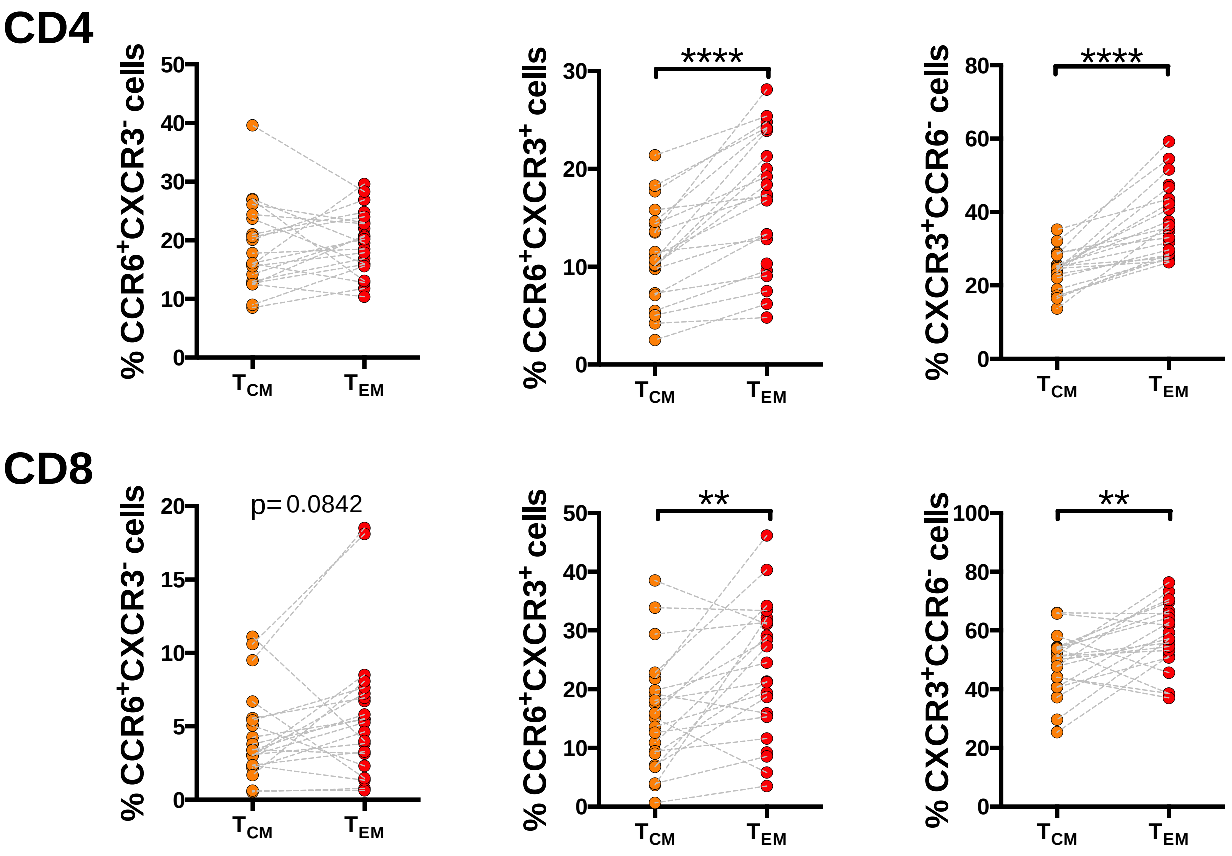

Supplemental figure 6

PBMCs isolated from healthy donors were stained for CD4 and CD8 lineage markers, memory T cell phenotype markers (CD45RA and CCR7) and for chemokine receptors CCR6, CXCR3. Statistical analysis of the differences was performed by Mann-Whitney test. p values <0.05 were considered significant: ** p<0.01, **** p<0.0001.

Ccr4

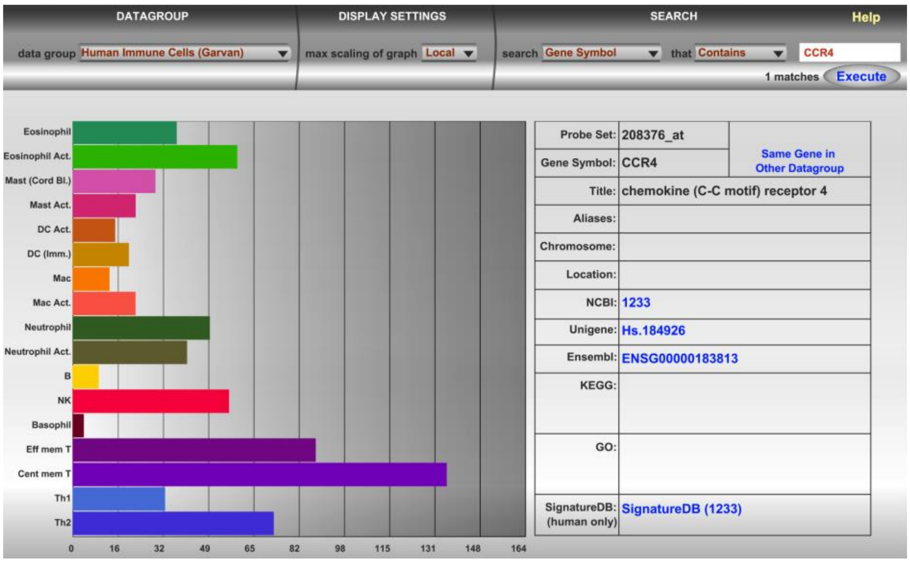

Ccr5

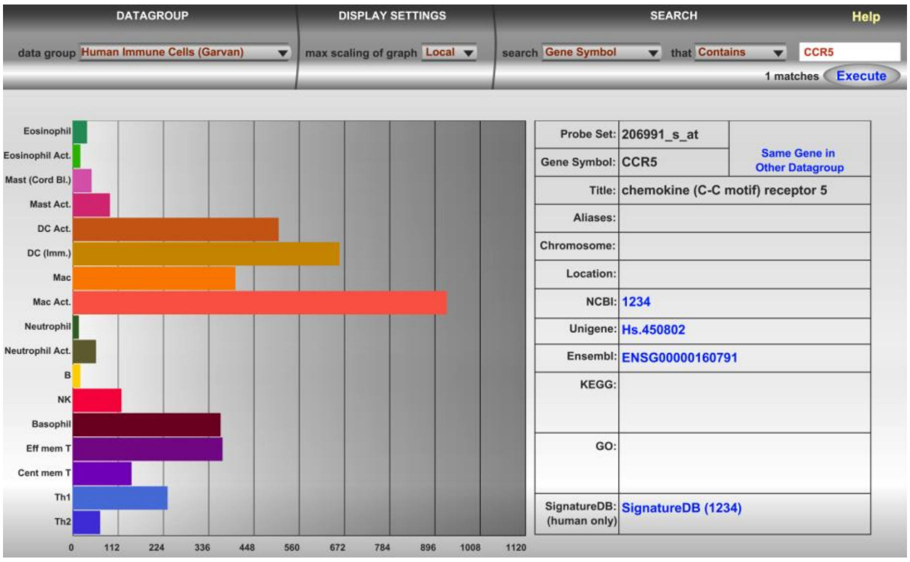

Supplemental figure 7

Gene expression data for *Ccr4* and *Ccr5* in individual immune cell population using ImmGen DataBrowsers (http://www.immgen.org/databrowser/index.html). Gene expression analysis was performed in Human Immune cells (Garvan) data group.

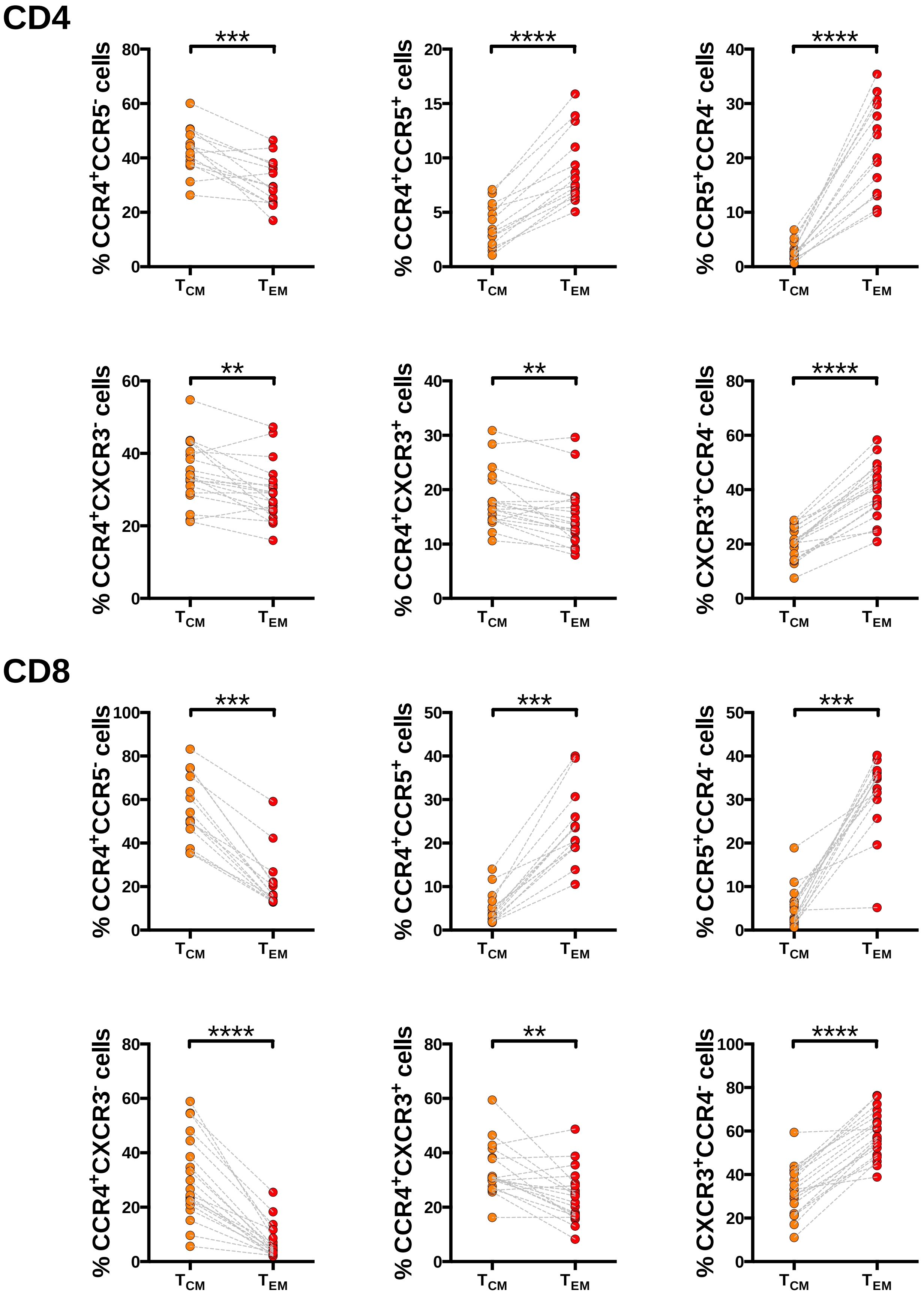
CD4
% CCR4+CCR5- cells
% CCR4+CCR5+ cells
% CCR5+CCR4- cells
% CCR4+CXCR3- cells
% CCR4+CXCR3+ cells
% CXCR3+CCR4- cells
CD8
% CCR4+CCR5- cells
% CCR4+CCR5+ cells
% CCR5+CCR4- cells
% CCR4+CXCR3- cells
% CCR4+CXCR3+ cells
% CXCR3+CCR4- cells
TCM
TEM
***
****
**

Supplemental figure 8

PBMCs isolated from patients with cutaneous psoriasis were stained for CD4 and CD8 lineage markers, memory T cell phenotype markers (CD45RA and CCR7) and for chemokine receptors CCR4, CCR5, CXCR3. Statistical analysis of the differences between T_{CM} and T_{EM} cells was performed by Mann-Whitney test. p values <0.05 were considered significant: ** p<0.01, *** p<0.001.
